# Ultrasound-triggered release of vancomycin from a novel spinal device: Antibiotic release and efficacy *in vivo*

**DOI:** 10.1016/j.ijpharm.2025.125276

**Published:** 2025-01-26

**Authors:** Lauren J. Delaney, Priscilla Machado, Ji-Bin Liu, Rachel Evans, Asia Winslow, Neil Zhao, Christopher K. Kepler, Rajkishen Narayanan, Teeto Ezeonu, Viren Soni, Gagan Kaushal, Rachel Hilliard, Thomas P. Schaer, Noreen J. Hickok, Flemming Forsberg

**Affiliations:** a Department of Radiology, Thomas Jefferson University, 132 S. 10^th^ Street, Main 10^th^ Floor, Philadelphia, PA 19107, USA; b Department of Orthopaedic Surgery, Thomas Jefferson University, 1015 Walnut Street, Suite 501, Philadelphia, PA 19107, USA; c Rothman Institute, 925 Chestnut Street, Philadelphia, PA 19107, USA; d College of Pharmacy, Thomas Jefferson University, 1025 Walnut Street, Suite 301, Philadelphia, PA 19107, USA; e Department of Clinical Studies, New Bolton Center, University of Pennsylvania School of Veterinary Medicine, 382 W Street Road, Kennett Square, PA 19348, USA

**Keywords:** Spine, Infection, Prophylaxis, Ultrasound triggered release

## Abstract

Post-surgical spinal infection occurs in up to 20 % of patients, despite aggressive peri-operative antibiotic treatments. To improve prophylaxis, we have designed and evaluated an ultrasound-activated prophylactic antibiotic release system to combat post-surgical bacterial survival. Polylactic acid (PLA) clips (1 cm^3^) were 3D-printed with an interior reservoir (0.8 cm^3^) for carrying drug payload, specifically vancomycin (VAN). Under IACUC approval, clips were surgically implanted into the spines of sheep (n = 9) and swine (n = 2) by removing the spinous process at several levels of the lumbar spine. In the sheep, clips were insonated and the interstitial wound fluid was collected to quantify the ultrasound-triggered VAN release. Uninsonated control sheep exhibited an average VAN concentration of 6.32 ± 5.99 μg/mL after 72 h, while ultrasound-triggered clips released significantly higher VAN concentrations at 72 h (22.98 ± 11.22 μg/mL, p = 0.033). In the swine, device efficacy against *Staphylococcus aureus* was evaluated. Insonated sites saw significant reduction in colony forming units (CFU) to 4.3 ± 3.2 CFU in the activated clips, compared to uninsonated controls where bacterial colonization was higher (2898 ± 1214 CFU, p = 0.017). Overall, these results demonstrate the ability to non-invasively release VAN from an implanted reservoir *in vivo*, and that this VAN release is effective in mitigating invading microbes in the wound site

## 1. Introduction

Back pain is one of the most common maladies afflicting adults worldwide (Wu et al., 2020; Hoy et al., 2012). Conservative treatments for back pain include physical therapy, and epidural injection with progression to minimally invasive discectomy or laminectomy (Omidi-Kashani et al., 2014). However, patients who have failed these minimally invasive treatments and continue to experience intractable back pain and radiculopathy become candidates for spinal fusion surgery (Omidi-Kashani et al., 2014). Spinal fusion aims to reduce back pain by applying a bone graft between unstable vertebrae, usually stabilized by spinal hardware to improve the bone fusion rate (Omidi-Kashani et al., 2014). Over the past 2 decades, elective spinal fusion cases have increased by over 275 % in the United States alone (Deng et al., 2021), with an estimated annual case rate of 400,000 and rising (Beschloss et al., 2021). However, despite aggressive *peri*-operative antibiotic regimens, bacterial infection remains a major clinical concern for these procedures, with up to 20 % of patients experiencing post-surgical infections (Moyano et al., 2020; Schömig et al., 2020; Delaney et al., 2023). Infection risk is directly related to the surgical approach and duration of the operation (Kim et al., 2014). Additionally, these procedures are often complicated by other patient factors, including age, immune compromise, obesity, and other comorbidities, which contribute to the overall risk of infection (Schömig et al., 2020; Kim et al., 2014).

To minimize infection risk, surgeons employ several procedures. One of the front-line defenses is application of intravenous antibiotics prior to the operation as a standard of care measure (O’Neill et al., 2011). Additionally, many surgeons place approximately 1–2 g of powdered vancomycin (VAN) into the surgical dead space surrounding the spinal hardware (roughly 1–2 cm^3^) immediately prior to wound closure (O’Neill et al., 2011; Francis and Mericli, 2022). These methods have resulted in a moderate reduction in infection rates, but infections still occur in up to 20 % of cases (Moyano et al., 2020; Schömig et al., 2020). Interestingly, these surgical site infections appear to be linked to the dermatological microbiome at the surgical site, as the microorganisms that locally populate the skin are also responsible for surgical site infections (Long, 2024). In fact, studies have shown that *Staphylococci* account for approximately 80 % of all spinal infections, with *Staphylococcus aureus* representing the most common microorganism responsible for spinal infections (Bhavan et al., 2010; Cebrián Parra et al., 2012; Legrand et al., 2001; Loibl et al., 2014; Oliveira et al., 2018; Foster et al., 2019). The remaining 20 % of spinal infections are attributed to *Streptococci*; *Enterococci*, and anaerobic microorganisms (Cebrián Parra et al., 2012; Legrand et al., 2001; Loibl et al., 2014). Prophylactic action against infection relies on achieving therapeutic, and even supra-therapeutic, antibiotic concentrations, with respect to the minimum inhibitory concentration (MIC) of the invading microbes. For example, according to the Clinical Laboratory Standards Institute (CLSI) and the European Committee on Antimicrobial Testing (EUCAST), the MIC for VAN-sensitive *S. aureus* is 2 μg/mL but can increase to greater than 16 μg/mL as resistance develops (Kest and Kaushik, 2019; Mancuso et al., 2021; Moses et al., 2020; Shariati et al., 2020).

Unfortunately, when the microbes are present as adherent, matrix-encased bacterial structures (biofilms), antibiotic sensitivity can decrease by as much as 1000X (Delaney et al., 2019; LuTheryn et al., 2020; Wolcott et al., 2008; Health, N.I.o. Research on Microbial Biofilms, 2002). The presence of spinal instrumentation itself represents a fertile surface for pathogens to colonize and form biofilms (Health, N.I.o. Research on Microbial Biofilms, 2002). Biofilms frequently adhere to solid surfaces, especially implanted hardware, often resulting in devastating infection that can complicate and prolong recovery with negative impacts on quality of life (LuTheryn et al., 2020; Health, N.I.o. Research on Microbial Biofilms, 2002; Delaney, 2020; Hall-Stoodley et al., 2004). In fact, according to the National Institutes of Health (NIH), biofilms are responsible for over 80 % of microbial infections in the body (Health, N. I.o. Research on Microbial Biofilms, 2002). Biofilms can lead to chronic, recalcitrant infection which often necessitates revision surgery to replace the contaminated hardware (Delaney et al., 2019; LuTheryn et al., 2020; Wolcott et al., 2008; Health, N.I.o. Research on Microbial Biofilms, 2002). However, the spinal site requires the stabilization provided by the fusion hardware and is unable to rely on antibiotic elution systems (i.e., antibiotic spacers) used in other joint sites, further complicating treatment of recalcitrant implant-associated spinal infection (Collins et al., 2008; Mok et al., 2009). To prevent this scenario, we and others focus on prevention as the best strategy; we seek to augment the existing clinical standard-of-care antibiotic prophylaxis to further decrease infection rates. We hypothesize that any pathogens that have survived the first-wave of prophylactic antibiotics can be eradicated by a second, spatiotemporal, post-surgical delivery of prophylactic antibiotics at supra-therapeutic concentrations within the spinal fusion site, thus reducing postoperative infection rates.

**Fig. 1.**
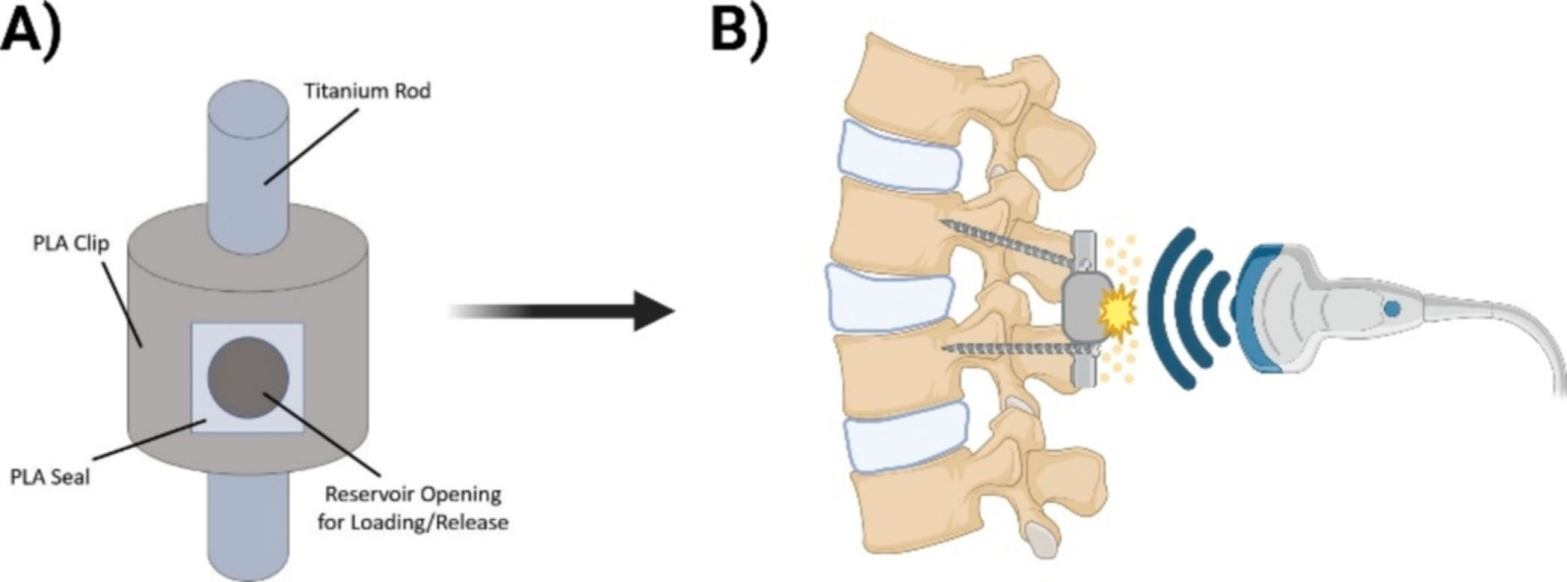
Schematic of clip and delivery system (not to scale). A) Diagram of clip with loading reservoir, PLA seal, and positioning on the spinal rod. B) Schematic of ultrasound-triggered rupture and payload release. Created with BioRender.com.

To this end, we have designed and evaluated an ultrasound-activated, spatiotemporal, release system to combat surgical site infections. Specifically, we have developed a polylactic acid (PLA) clip that is designed to attach onto standard titanium spinal fusion rods and serve as a non-elution reservoir of prophylactic antibiotics (Delaney et al., 2023; Delaney et al., 2019; Delaney et al., 2020). At the surgeon’s discretion, the reservoir is activated by application of transcutaneous ultrasound between 3–5 days following surgery when the first-wave of prophylactic antibiotics are depleted, and surgical drains are removed (Zhao et al., 2024). Ultrasound causes rupture of the thin PLA film sealing the reservoir, aided by the inclusion of microbubbles within the antibiotic solution to serve as additional cavitational nuclei. The ultrasound-triggered film rupture within the surgical site triggers a localized release of supra-therapeutic antibiotic concentrations, designed to far exceed the MIC. A representative diagram of this delivery system is shown in Fig. 1.

The purpose of this study was to perform *in vivo* evaluations of this ultrasound-triggered delivery system. First, the ultrasound-triggered release of VAN from within the clips into the interstitial wound fluid was quantified in a sheep model of spinal surgery to test stability and activation functionality of these devices. Then, we evaluated the efficacy of this concept against common bacteria, specifically *Staphylococcus aureus*, in a swine model of spinal surgery. Both ovine and porcine spines are widely considered good models for the human spine, supporting their use in this study (Wilke et al., 1997; Sheng et al., 2010; Mageed et al., 2013; Busscher et al., 2010; Yingling et al., 1999).

## 2. Materials & methods

All animal studies were performed under Institutional Animal Care and Use Committee (IACUC) approval at our institutions and followed Animal Research: Reporting of *In Vivo* Experiments (ARRIVE) guidelines. Unless otherwise noted, laboratory supplies were acquired from Fisher Scientific (Hampton, NH, USA).

### 2.1. Ultrasound-triggered polymer clip preparation

The C-shape design of the PLA clips was specifically chosen to stably attach onto the standard spinal fusion hardware, specifically the titanium stabilizing rod. The clip design included an inner drug reservoir, for encapsulating the payload, as described previously (Delaney et al., 2023; Delaney et al., 2019; Delaney et al., 2020). Briefly, these PLA clips (1.0 cm^3^) were 3D-printed using a 2.85 mm, 750 g filament (Ultimaker, Utrecht, The Netherlands). The volume of the entire clip is 0.8 cm^3^, with an interior hollow reservoir able to contain therapeutically-relevant volumes (up to 0.25 cm^3^) of the prophylactic drug payload. The curved outer surface of the clip was designed with a 4-mm circular opening for drug loading and subsequent ultrasound-triggered release (c.f., Fig. 1).

VAN was chosen as the drug payload, since it is one of the most commonly used prophylactic antibiotics in clinical practice. A VAN suspension (Athenex, Buffalo, NY, USA) with a final concentration of 400 mg/mL was prepared by reconstituting 1 g powdered VAN in 2.5 mL sterile distilled water. Sterile water was used as the suspension medium to simulate clinical re-suspension of powdered VAN. Clips were loaded with approximately 180 μL of the VAN suspension and 50 μL of Sonazoid microbubbles (GE HealthCare, Oslo, Norway), for a total VAN loading of 72 mg (final concentration of 313 mg/mL, corresponding to ~ 1500x the MIC of VAN-sensitive *S. aureus*). The Sonazoid microbubbles were reconstituted in sterile water per manufacturer’s instructions and were included in the clip reservoir to act as cavitation nuclei to aid in the rupture of the PLA film seal releasing the housed VAN. Following loading, the clips were sealed with a hand-cast PLA (Resomer Select 100 DL 7E; Evonik Industries, Essen, Germany) film (0.05 ± 0.01 mm thick) secured to the clip by brushing with chloroform. Sealed clips were dried under continuous airflow at room temperature for 24 h in a laminar flow hood to maintain sterility.

### 2.2. Evaluation of ultrasound-triggered rupture and VAN release in a sheep model

Loaded, sealed clips were surgically implanted into 9 skeletally mature, healthy, female Dorset crossbred sheep (~70 kg mean weight, Archer Farms, Darlington, MD, USA) along the spinal midline by an orthopedic surgeon, who removed the spinous process at the L2 (cranial) and L5 (caudal) levels for inserting the clips (2 sites per sheep, n = 18 sites). Gender was kept singular (female) to limit biological variability. Briefly, under endotracheal anesthesia using isoflurane gas inhalation mixed with oxygen, the surgical site was draped and prepared for surgery. The dorsal spinous processes were exposed via a curvilinear incision at the desired spinal level, then the dorsal spinous process was resected, and the clip was snuggly implanted and sutured into place in the resulting dead space. Ultrafiltration probes (RUF-3–12 Reinforced Ultrafiltration Sampling Probes, Bioanalytical Systems, Inc., West Lafayette, IN, USA) were also placed into each wound site prior to closure for collection of interstitial wound fluid and detection of VAN release from the clips into the wound space. Following recovery from anesthesia, sheep were returned to their respective vivarium with unrestricted access to food and water as well as exercise in a 12′ by 12′ stall with natural bedding for 72 h, with fluid collections taken from the ultrafiltration probes every 24 h until further intervention depending on group assignment.

Three of the sheep were used as controls to assess the longevity and stability of the clips and the payload encapsulation within the clips in the absence of ultrasound. These animals were euthanized following the final ultrafiltration probe collection at 72 h post-implantation surgery, with the clips and surrounding tissues harvested for analysis.

The remaining 6 sheep were assigned to the experimental group that received ultrasound triggering of the clips. Seventy-two hours post-operative, the sheep were re-anesthetized for insonation in sternal recumbency. Each site was insonated for 20 min using a Logiq E9 clinical ultrasound scanner (GE HealthCare, Waukesha, WI, USA) equipped with a C1–6 curvilinear probe in power Doppler mode (1.7 MHz central frequency, 5.4 kHz pulse repetition frequency, 100 % acoustic output power, mechanical index 1.2, equating to approximately 146 mW/cm^2^ I_SPTA_ intensity) (Delaney et al., 2020), to rupture the PLA seal and induce bolus release of the encapsulated payload. The insonation was performed by a radiologist (PM) with over 15 years of experience with humans and animal models. Following insonation and still under general anesthesia, the sheep were then placed into dorsal recumbency for 30 min to simulate human positioning during recovery. Then, sheep were recovered from anesthesia and returned to their respective vivarium for unrestricted exercise. Ultrafiltration probe collections were taken every hour for the first 6 h, then again at 12, 24, 48, and 72 h. After 72 h post-insonation (6 days post-implantation), the animals were euthanized, with the clips and surrounding tissues harvested for analysis.

After sacrifice and clip retrieval, surgical sites and clips were photographed and assessed for visual signs of activation (i.e., visible disruption of the PLA film). Any clips that appeared to have an intact PLA film, including the uninsonated control clips, were manually compromised via needle puncture and peeling, and the fluid remaining inside the clips was aspirated via pipette for analysis with UV–Vis (Nanodrop, Thermo Fisher Scientific, Waltham, MA, USA).

Ultrafiltration probe collections were analyzed with liquid chromatography/mass spectrometry (LC-MS), using 1260 Agilent Infinity II and 6545 XT LC/QTOF (Agilent Technologies, Inc., Santa Clara, CA, USA). All scans were performed under positive ion mode and electron spray ionization (ESI), in the scanning mass range of 200–1500 *m*/*z*. Experimental parameters were optimized using MassHunter Workstation software (Agilent) as such: sheath gas flow rate of 75.8 kPa, auxiliary gas flow of 34.5 kPa, electron spray voltage of 3.50 kV, capillary temperature of 375 °C with capillary, tube lens, and skimmer voltage kept at 67.5 V, 130 V, and 28 V, respectively. LC-MS runs were performed using isocratic elution with 60:40 (water with 0.1 % formic acid: acetonitrile with 0.1 % formic acid). injection volumes were set to 5 μL and chromatographic separations were performed using XBridge^®^ HILIC, 3.5 μm (4.6 × 100 mm) column (Waters, Milford, MA, USA) at a flow rate of 0.35 mL/min with a run time of 5 min. Column compartment and sampler temperature was set to 30 °C and 20 °C, respectively. Primary VAN stock calibration curve was made from 100–2000 ng/mL in LC-MS grade water, made by spiking blank sheep wound fluid to final concentrations of 50–1000 ng/mL. Extraction of VAN (from calibration spike standards and samples) was performed using the protein precipitation method, with sheep wound fluid and acetonitrile at a 1:2 ratio. The lower limit of detection and quantitation were found to be 1 ng/mL and 10 ng/mL, respectively.

### 2.3. Evaluation of ultrasound-triggered VAN release efficacy against bacteria in a swine model

**Fig. 2.**
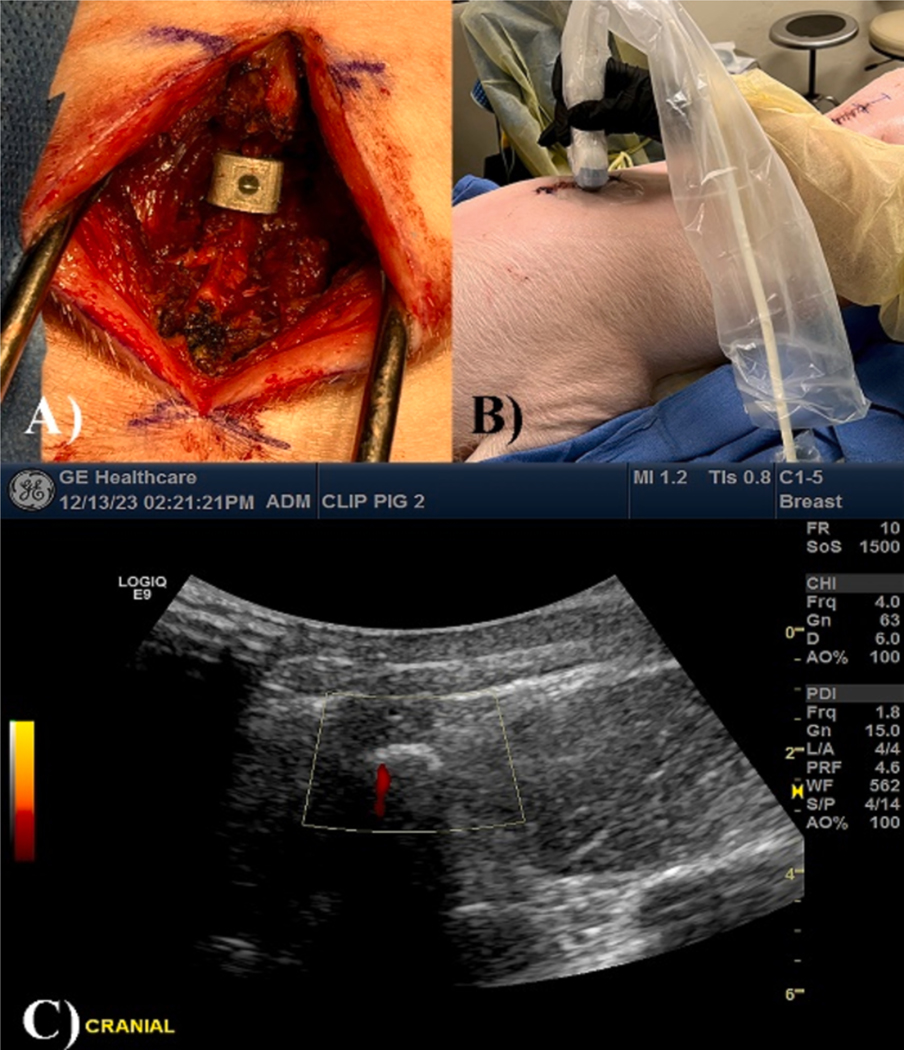
Representative images of experimental process. A) Representative image of clip implantation into the spinous process at the L1 level. B) Image of insonation of the wound site following bacterial inoculation and wound closure. C) Representative ultrasound image showing insonation of the clip (within the yellow box).

Similar to the implantation procedures described previously for the sheep, clips were surgically implanted into 2 female Yorkshire pigs (~50 kg each, Animal Biotech Industries, Doylestown, PA, USA) along the spinal midline by an orthopedic surgeon. Gender was kept singular (female) to limit biological variability. Three clips were implanted into the spinous process space at approximately the T8, L1, and L5 levels (3 sites per pig, n = 6 sites total). A representative image of the clip implantation is shown in Fig. 2A. Prior to wound closure, 10^6^ colony forming units (CFU) of *Staphylococcus aureus* (*S. aureus*, ATCC 25923, Manassas, VA, USA) in phosphate buffered saline (PBS) were introduced to the wound sites. The *S. aureus* were cultured overnight in PBS at room temperature on a shaker plate, then quantified via microscopy and diluted with PBS to reach the desired concentration. The wounds were sutured in layers, changing suture and gloves upon conclusion of each site to prevent any cross-contamination.

The middle site (~L1) in each swine was used as an uninsonated control (n = 2), allowing each swine to serve as its own biological control for bacterial survival, propagation, and colonization. Each remaining experimental wound site (n = 4) was insonated transdermally as described previously (representative images shown in Fig. 2B and Fig. 2C). Swine were then recovered from anesthesia and allowed unrestricted food, water, and exercise in a 12′ by 12′ stall with natural bedding. Twenty-four hours post-insonation, the animals were euthanized, with the clips and surrounding tissues harvested for analysis. After sacrifice and clip retrieval, surgical sites and clips were photographed and assessed for visual signs of activation (i.e., visible disruption of the PLA film). Additionally, bacterial colonization was assessed by rolling the retrieved clips and swabs of the wound sites onto agar plates, with bacterial counts analyzed after 24- and 72-hours incubation at 37 °C with 5 % carbon dioxide and standard water bath for humidity. No additional nutrients or media were added to the agar plates during the incubation so that natural growth could occur. Bacterial colonies were hand-marked for counting, then the marked colonies were counted with ImageJ software (NIH, Bethesda, MD, USA).

### 2.4. Statistical analysis

All statistical testing was performed using Prism 10 (GraphPad, San Diego, CA, USA) using α = 0.05 significance level. Comparison of VAN release between time points was performed using paired Student’s t-tests, while comparisons against controls were performed using unpaired Student’s t-tests. Comparison of bacterial counts was performed using unpaired Student’s t-tests. When appropriate, Bonferroni correction was applied for multiple comparisons. Data is reported as the mean ± standard deviation (SD) as appropriate.

## 3. Results

All animals recovered uneventfully from the implantation and insonation procedures, with no adverse effects. All procedures were performed under IACUC-approved protocols at both Thomas Jefferson University and University of Pennsylvania School of Veterinary Medicine. All euthanasia was performed consistent with the American Veterinary Medical Association (AVMA) guidelines.

### 3.1. Evaluation of Ultrasound-Triggered rupture and VAN release in a sheep model

First, we performed control experiments by evaluating five clips in 3 sheep that did not receive subsequent insonation, with 2 sheep having both the cranial (L1) and caudal (L5) sites implanted, and 1 sheep implanted at only the cranial level (cranial n =3, caudal n =2). Seventy-two to 96 h post-implantation, sheep were euthanized, and clips were excised for analysis.

The PLA films on all the control clips (uninsonated) appeared intact upon visual inspection after explantation. Following needle puncture of the films, the aspirated fluid was clear in nature, similar in appearance to the initial loading solution. The UV–Vis analysis of the aspirated fluid determined that the concentration of both the cranial clips (295.10 ± 36.31 mg/mL) and the caudal clips (270.40 ± 20.34 mg/mL) were similar to each other (p = 0.76), and also similar to the initial loading solution (313.00 ± 20.00 mg/mL, p = 0.27). There is the possibility of mild dilution of the aspirated fluid from within the clips due to bodily fluid exchange *in situ* and fluid extraction techniques post-explantation. However, the retention of fluid concentrations suggests that bodily fluid was not exchanged between the interior of the clip reservoir and the wound site in large quantities in the absence of insonation.

Interestingly, the LC-MS quantification of the wound fluid surrounding these control clips further elucidated the fluid exchange and device stability in the wound sites during the up-to-96-hour incubation between implantation and explantation. In the cranial site, VAN concentrations in the wound fluid ranged from 50.10 ng/mL to 18.29 μg/mL, with an average of 6.32 ± 5.99 μg/mL. Concentrations at the caudal site were higher with an average of 151.88 ± 0.11 μg/mL; albeit not statistically significant (p = 0.09), suggesting this site was more prone to premature leakage from the device due to shear stresses at this anatomical location (Keller et al., 2005). Therefore, we chose to exclude the caudal site from further analysis and focus on the more stable cranial site. These findings suggest that the clips in the cranial site maintain their integrity *in vivo* for at least 5 days post-operatively in the absence of insonation, aligning with the desired delivery mechanism.

**Fig. 3.**
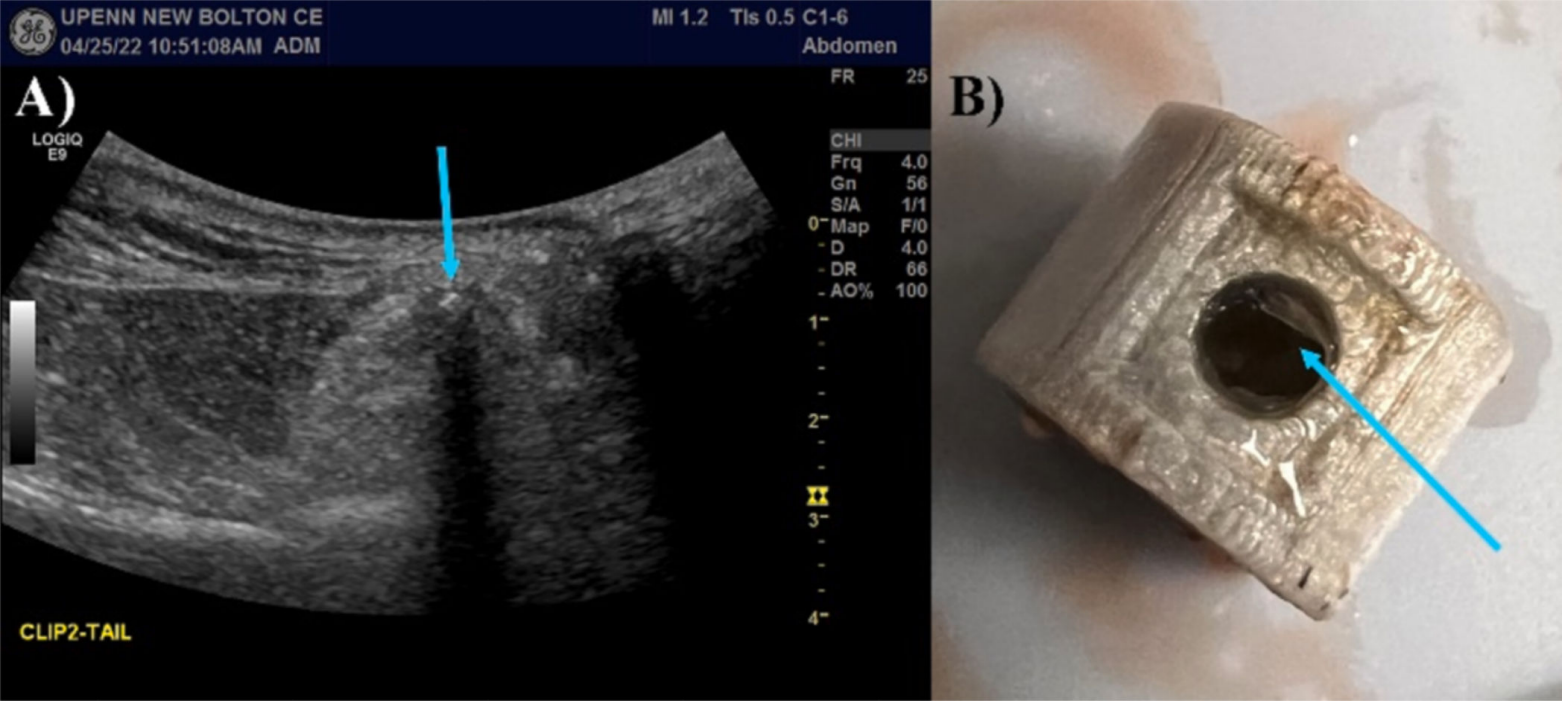
Representative images from insonation. A) Representative ultrasound image showing visualization of the clip (blue arrow) in the spinal space. B) Representative image of a retrived ultrasound-ruptured clip, blue arrow shows site of film rupture.

Turning to the sheep that received insonation, all the PLA film seals (6/6 at the cranial site and 6/6 at the caudal site, 100 %) appeared ruptured, some partially and some completely, upon harvest and visual inspection. Representative images of the ultrasound triggering of the implanted device and a retrieved clip are show in Fig. 3. Small amounts of fluid collected from the inside of the clips appeared bloody and cloudy in nature, further supporting film compromise with fluid exchange. UV–Vis was not performed on these reservoir contents, due to the overlapping absorbance of the bodily fluids. However, the LC-MS analysis of the surrounding wound fluid provided confirmation of the release of VAN from within the clips into the wound space. Over the 72-hour incubation period between implantation and insonation, an average of 7.51 ± 5.86 μg/mL was measured in the wound exudate from the cranial site, which is similar (p = 0.90) to the baseline release at the cranial site measured in the uninsonated control sheep.

**Fig. 4.**
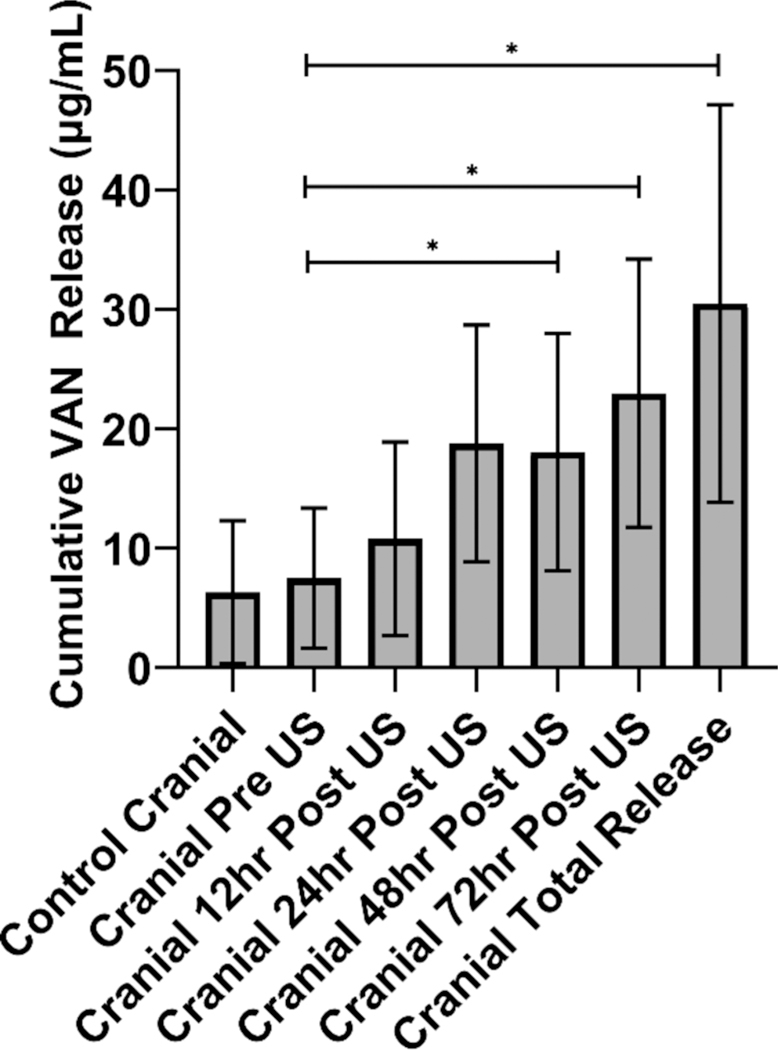
Graph of cumulative VAN release over time, n = 3 for control, n = 6 for experimental groups, error bars represent standard deviation, *p < 0.05.

Cumulative VAN release at the cranial site following insonation was generally greater compared to pre-insonation baseline release levels, as shown in Fig. 4 and Supplementary Fig. 1. Cumulative ultrasound-triggered VAN release at 12 h post-insonation (10.80 ± 8.09 μg/mL, ~5x MIC for VAN-sensitive *S. aureus*, p = 0.11) was not significant, while at 24 h post-insonation (18.78 ± 9.90 μg/mL, ~9x MIC, p = 0.057) it was trending toward significance. Significance was reached at 48 h post-insonation (18.06 ± 9.94 μg/mL, ~9x MIC, p = 0.049) and 72 h post-insonation (22.98 ± 11.22 μg/mL, ~11x MIC, p = 0.033) post-insonation. Overall, clips released an average of 10.10 ± 5.00 μg/mL over the first 24 h post-insonation (and 15.46 ± 6.58 μg/mL over 72 h post-insonation), achieving the desired ultrasound-triggered bolus release at approximately 8x the MIC for VAN-sensitive *S. aureus*.

These results demonstrate the ability to produce a percutaneous ultrasound-triggered rupture of the thin PLA film encapsulating the prophylactic solution within the clip device in all 12 insonated clips (12/12 or 100 %). Additionally, we have demonstrated the stability and integrity of 5 control clips in the postoperative cranial (~L1) surgical site for up to 96 h without exposure to ultrasound, retaining the majority of the VAN solution within the device until the PLA film was ruptured. Furthermore, we did find that there were differences between the two anatomical locations along the ovine spinal column, with the cranial site providing more stability than the caudal site.

### 3.2. Evaluation of ultrasound-triggered VAN release efficacy against bacteria in a swine model

**Fig. 5.**
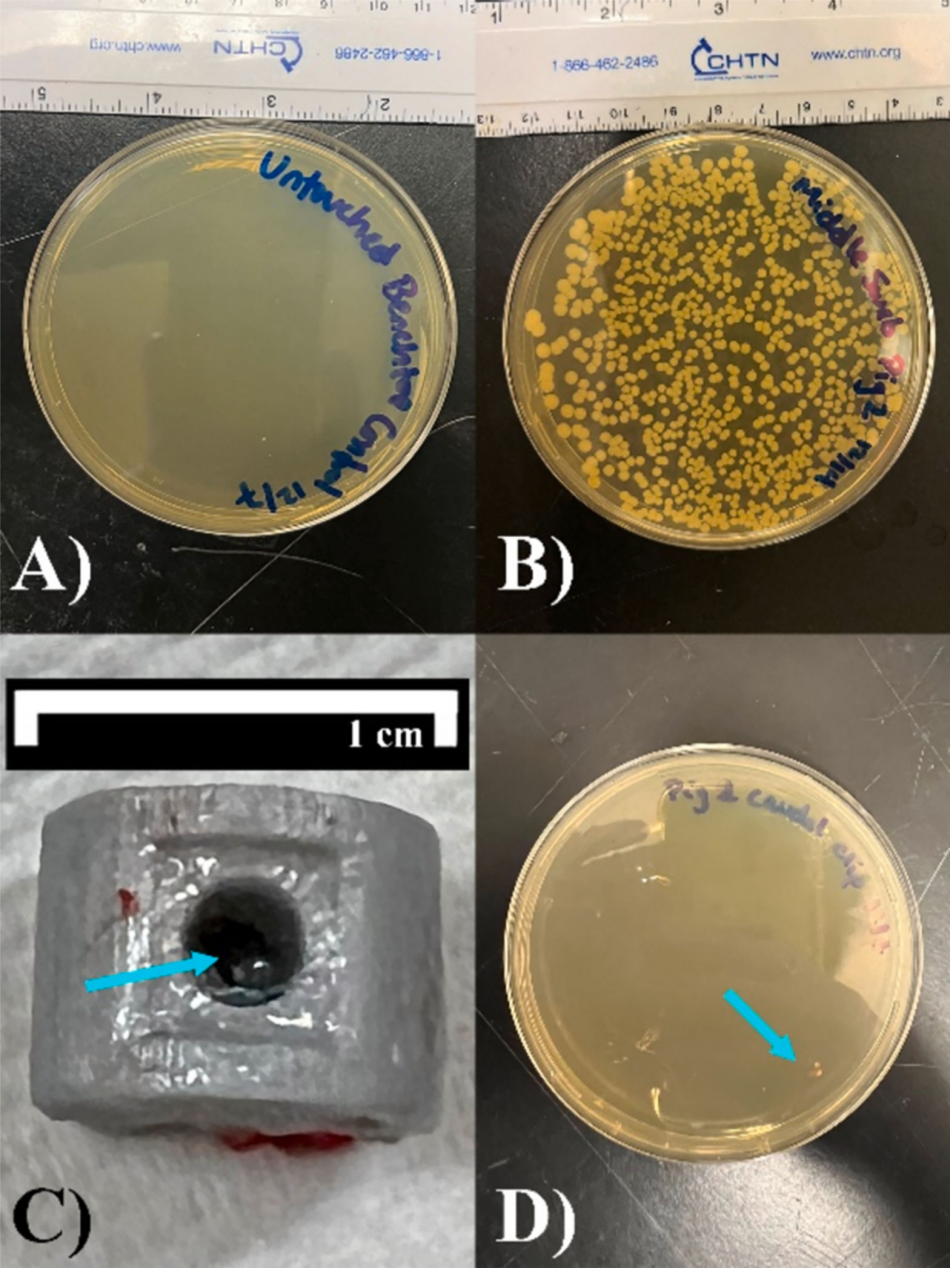
Representative images of bacterial culture after 72 h incubation and post-US clip rupture. A) Culture of benchtop control plate showing no growth. B) Culture of uninsonated wound site showing prolific bacerial growth. C) Representative image of US-ruptured clip, blue arrow showing visible rupture of PLA film over reservoir. D) Culture of clip surface from insonated site showing minimal bacterial growth (blue arrow).

Similar to what was observed in the sheep, the PLA film seals on the uninsonated clips in the swine (n = 2) showed no visible signs of rupture or release. Additionally, both the excised clips and the wound sites exhibited abundant bacterial growth after both 24 h of incubation (2921 ± 1200 CFU) and after 72 h of incubation (2898 ± 1214 CFU), as shown in Fig. 5B. Moreover, the clip and wound site from sheep #1 resulted in a bacterial lawn (> 5000 CFU) at both 24 and 72 h, while the clip and wound site from sheep #2 were lower (883 and 802 CFU, respectively), highlighting the importance of this experimental design with each animal serving as its own biological control for bacterial growth (cf., Table 1). As a control, agar plates that were left open to the environment in the surgical suite resulted in 0.0 ±0.0 CFU growth at both 24 and 72 h of incubation (Fig. 5A), suggesting that the cultures observed at harvest were a result of the bacterial inoculation of the wound and not environmental contamination.

Importantly, following insonation, 3 of the 4 insonated clips were visibly ruptured, with the fourth clip (swine #1 cranial site) showing no signs of rupture or release. A representative image of an excised ultrasound-ruptured clip is shown in Fig. 5C. As shown in Table 2, swabs of the clips and wound sites that ruptured in response to ultrasound yielded little to no bacterial growth, with only 4.3 ± 3.2 CFU present after 72 h of incubation (Fig. 5D), signifying a significant reduction in bacterial number compared to controls (p = 0.017). On the other hand, swabs of the one insonated but non-ruptured clip (swine #1 cranial site) resulted in colonies too numerous to count (i.e., a “lawn” of bacteria) at both 24 and 72 h (cf., Table 2), similar to that of the uninsonated controls (in Table 1, p = 0.46). The non-ruptured clip also exhibited a slight reduction in CFU at 72 h, which is due to increased colony size leading to merging of neighboring colonies, reducing the overall individual counts.

These results demonstrate the integrity of the VAN activity during encapsulation, implantation, and ultrasound triggering *in vivo*. We observed significant reduction in bacterial load following ultrasound-triggered release, compared with the sites that did not experience ultrasound-triggered release.

## 4. Discussion

Overall, these results demonstrate the ability to produce a percutaneous ultrasound-triggered rupture of a thin PLA film sealing a clip device to cause a significant VAN release *in vivo*. First, the stability and integrity of the clips was demonstrated in the cranial site of the sheep, and again in the middle control site of the swine. The results demonstrated that the concentrations of released VAN in the wound sites, as measured by LC-MS analysis of the sheep wound fluid, aligns with the observed antibacterial activity in the swine. These findings suggest that the VAN remained active, while encapsulated within the device, and was not compromised by the insonation. In fact, we and others have shown that *in situ* ultrasound can enhance antibiotic activity against both planktonic bacteria and biofilms (Zhao et al., 2023; Carmen et al., 2004; He et al., 2011).

The choice of VAN as the payload in these clips was guided by *S. aureus* being identified as one of the main invading microbes responsible for implant-associated infection (Oliveira et al., 2018; Foster et al., 2019). Currently, antibiotic levels are described and estimated by the MIC, which is heavily dependent on the sensitivity of the microbe and is derived from planktonic bacteria. For example, CLSI and EUCAST guidelines suggest that the MIC for VAN against *S. aureus* ranges from ≤ 2 μg/mL for sensitive phenotypes to > 16 μg/mL for resistant phenotypes (Kest and Kaushik, 2019; Mancuso et al., 2021; Moses et al., 2020; Shariati et al., 2020). Additionally, recent discussion has centered on whether MIC is an effective measure of efficacy, or whether other more comprehensive measures such as the minimum concentrations for biofilm inhibition (MBIC) and biofilm eradication (MBEC) should also be considered (Zhao et al., 2023; Carmen et al., 2004). Nonetheless, we have demonstrated that these clips release ≥ 100 μg/mL VAN *in vitro* (~50x MIC for sensitive phenotypes) (Delaney et al., 2023; Delaney et al., 2019), and up to ~ 23 μg/mL VAN *in vivo* (~11.5x MIC) to the area surrounding the device. As such, the release profile from these clips is amenable to effective prophylaxis against any remaining invading microbes in the wound site as a second-wave treatment adjunctive to the clinical standard of care.

This delivery system was designed based on the hypothesis that a second, spatiotemporal dose of prophylactic antibiotics could effectively eradicate any intra-operative microbial contamination that eluded the first-wave perioperative treatments (Delaney et al., 2023; Delaney et al., 2019; Dastgheyb Sana, 2015; Lewis et al., 2008). Additionally, the system is designed so that the payload could be either a single or combination of antibiotics, active against both Gram-positive and Gram-negative pathogens, for broad-spectrum coverage of potential invading microbes in the wound site while avoiding the problems of highly variable elution kinetics of conventional elution systems (Delaney et al., 2023; Delaney et al., 2019; Delaney et al., 2020). Importantly, by delaying the payload delivery by approximately 3 days post-surgery, any bacterial colonies that remain in the wound site have time to transition from tolerance to the first-wave treatment into a phenotype that is more antibiotic-susceptible (Dastgheyb Sana, 2015; Lewis et al., 2008; Cheng et al., 2024). Additionally, the delayed bulk release profile is preferred compared to a slow, uncontrolled elution, which could induce selective pressure and create an environment for developing antibacterial tolerance (Dastgheyb Sana, 2015; Lewis et al., 2008; Cheng et al., 2024; Gao et al., 2022). In keeping with this, current consensus is that the powdered antibiotics that are *peri*-operatively placed into the wound site are depleted within the first 24–48 h following surgery (unpublished data), mainly due to the presence of aseptic wound drains (Delaney et al., 2019); where our device would allow for a 24–48 h break from bacterial challenge once these peri-operative antibiotics are depleted before providing the second bolus dose. In fact, we have demonstrated the possibility of this occurring as part of this study, where the ultrafiltration probes collecting the VAN-laden wound fluid mimicked the action of the aseptic wound drains, albeit on a smaller scale. Importantly, the VAN payload was retained within the majority of the implanted clips prior to insonation, while the insonated clips resulted in reduction of the bacterial bioburden in the wound site below our detection sensitivity, while the unruptured clips did not.

It is important to note that our drug delivery system is meant to release second-wave prophylactics, aimed at preventing colonization of the spinal hardware and development of biofilms and recalcitrant infection from those bacteria that survived the first-wave prophylaxis. Once a surgical site infection has become established, systemic intravenous antibiotics are often ineffective, necessitating aggressive revision surgeries with debridement and hardware modification or replacement (Dowdell et al., 2018; Lener et al., 2018). Unfortunately, in the spine, there are relatively few interventions available to clinicians aside from aggressive debridement and hardware replacement, with the application of antibiotic-loaded bone cement as the most common method of hardware modification (Masuda et al., 2017; Chiu et al., 2024; Chen et al., 2024; Kuris et al., 2023). While this method has had some success in treating established infections, it is invasive and results in prolonged pain and suffering for the patients. Therefore, our second-wave prophylactic system, which aims to prevent development infection from establishing, while also preventing the need for revisions which reopen the wound and increase the chances of introducing additional invading microbes, has potential for significant clinical impact.

While the study findings presented in this paper are encouraging, there are several limitations that necessitate discussion. One limitation to the study was the lack of stability at the caudal site in the sheep, which limited the use of the wound fluid samples and controls. However, we believe that the observed integrity of the uninsonated clips at the cranial sheep site, as well as in the swine model, provides appropriate evidence that the PLA film would not be structurally or functionally compromised by these elements in biologically-relevant models with spinal instrumentation to provide additional stability (Wilke et al., 1997; Sheng et al., 2010; Mageed et al., 2013). We appreciate that the uninsonated clips were only incubated in the swine for 1 day, while they were incubated within the sheep for 3 days, but the stability of the control clips in both species further supports this degree of integrity in a bipedal human. Another important limitation to this study was the small sample size for the *in vivo* evaluations. Following the 3 Rs of responsible *in vivo* research, we performed this pilot study using sheep and swine based on the results of our previous studies (Delaney et al., 2023). Sheep and swine are frequently used in spinal surgery studies, and thus afforded us a realistic surgical site including substantial amounts of dead space akin to a surgical site in a human patient (Ward et al., 2017). We were also limited by inconsistent wound fluid production and subsequent collection in the ultrafiltration probes over the 6-day sheep study, reducing the available data points for several of the animals. As the study progressed, we added additional ultrafiltration probes to the wound site in an effort to rectify the collection issues. We also acknowledge that the bacterial culture experiments were performed using simple agar plate culturing methods, and used only planktonic bacteria in the wound site. However, we expect our prophylactic delivery system to be addressing mostly planktonic bacteria in the wound site, since it is intended to be ultrasound-activated before bacteria can become adherent and form biofilms. Given our encouraging proof-of-concept findings, we are confident that we have established sufficient translational merit in support of continued developments towards clinical utility. Finally, we acknowledge that next steps have to include experiments demonstrating efficacy using a bacterial infection challenge model in the presence of spinal hardware, both *in vitro* and *in vivo*. This will be necessary to fully characterize the active antibiotic release profile and to demonstrate effective biologically-relevant bacterial reduction using this technology, acknowledging that in our *in vivo* studies complete independence between surgical sites is assumed but cannot be fully guaranteed. Despite these limitations, the results of this study justify continued exploration of this physician-activated, ultrasound-triggered prophylactic delivery system aimed at mitigating early bacterial colonization of spinal hardware.

## 5. Conclusions

Existing methods are only partially successful in preventing postoperative infection after spinal fusion surgery (which occur in up to 20 % of patients). To combat this problem, we have designed an ultrasound-activated drug release system to carry combinations of prophylactic antibiotics, while avoiding the problems of highly variable elution kinetics of conventional elution systems. We evaluated the efficacy of these devices against bacteria in an *in vivo* swine model of spinal surgery, achieving significant reductions in bacterial colony counts observed following ultrasound activation. We also showed agreement between quantitative vancomycin release from an *in vivo* sheep study with the observed antibacterial activity in the swine. There was significant vancomycin release over 12 to 72 h post-insonation, indicating effective ultrasound-triggered bolus release. These proof-of-concept *in vivo* results are encouraging and support continued evaluation of these devices aimed at mitigating early bacterial colonization of spinal hardware and reducing the risk of devastating perioperative, implant-associated infection.

## Supplementary Material

Supp.fig1

## Figures and Tables

**Table 1:** Average CFU (± SD) following culture of retrieved clips and wound site swabs.

	Insonated & 24 hr Incubation (n = 4)	Insonated & 72 hr Incubation (n = 4)	Uninsonated & 24 hr Incubation (n = 2)	Uninsonated & 72 hr Incubation (n = 2)

**Clips**	1028 ± 1027 CFU	953.3 ± 952 CFU	2942 ± 2059 CFU	2915 ± 2086 CFU
**Wound Sites**	1254 ± 1249 CFU	1255 ± 1248 CFU	2901 ± 2099 CFU	2882 ± 2119 CFU
**Overall**	1141 ± 750 CFU	1104 ± 729 CFU	2921 ± 1200 CFU	2898 ± 1214 CFU

**Table 2:** Average CFU (± SD) of insonated clips and wound sites.

	US-Ruptured & 24 hr Incubation (n = 3)	US-Ruptured & 72 hr Incubation (n = 3)	Non-Ruptured & 24 hr Incubation (n = 1)	Non-Ruptured & 72 hr Incubation (n = 1)

**Clips**	0.7 ± 0.3 CFU	1.7 ± 0.8 CFU	4109 CFU	3808 CFU
**Wound Sites**	5.0 ± 5.0 CFU	7.0 ± 6.5 CFU	5000 CFU	5000 CFU
**Overall**	2.8 ± 2.4 CFU	4.3 ± 3.2 CFU	4555 ± 446 CFU	4404 ± 596 CFU

## Data Availability

Data will be made available on request.

## Appendix A. Supplementary data

Supplementary data to this article can be found online at https://doi.org/10.1016/j.ijpharm.2025.125276.
