# Supplementary figures and images for: Ultrasound-triggered release of vancomycin from a novel spinal device: Antibiotic release and efficacy in vivo
Source: Int J Pharm. Author manuscript; Available in PMC 2026 Jun 22. (PMC13285313; doi:10.1016/j.ijpharm.2025.125276)

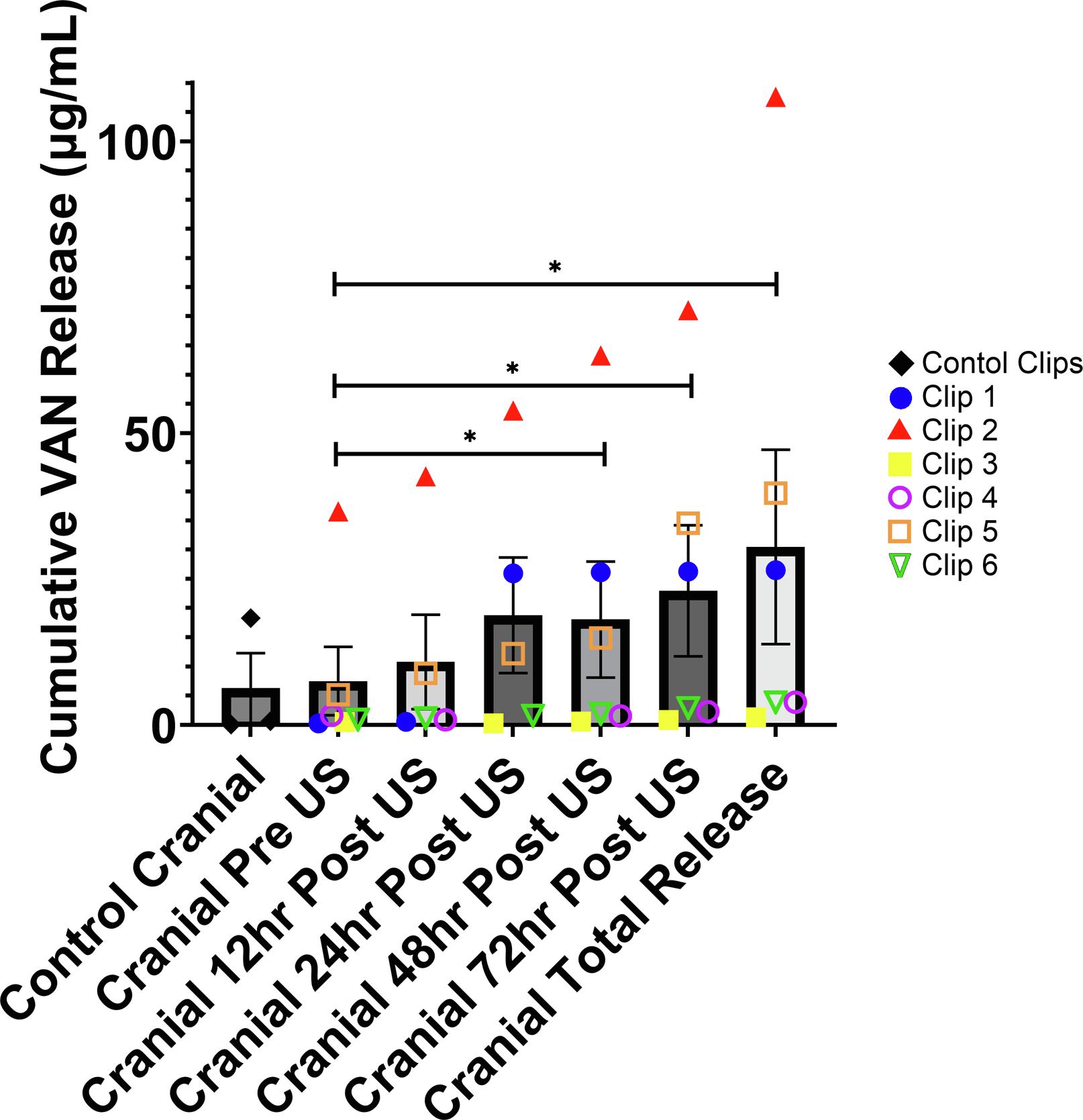

Supplement: Supp.fig1 [file NIHMS2171285-supplement-Supp_fig1.jpg]
